# Favorable pleiotropic effects of sodium glucose cotransporter 2 inhibitors: head-to-head comparisons with dipeptidyl peptidase-4 inhibitors in type 2 diabetes patients

**DOI:** 10.1186/s12933-020-0990-2

**Published:** 2020-02-12

**Authors:** Shih-Chieh Shao, Kai-Cheng Chang, Swu-Jane Lin, Rong-Nan Chien, Ming-Jui Hung, Yuk-Ying Chan, Yea-Huei Kao Yang, Edward Chia-Cheng Lai

**Affiliations:** 1 Department of Pharmacy, Keelung Chang Gung Memorial Hospital, Keelung, Taiwan; 2 School of Pharmacy, Institute of Clinical Pharmacy and Pharmaceutical Sciences, College of Medicine, National Cheng Kung University, No. 1, University Road, Tainan, 701 Taiwan; 3 Department of Pharmacy, Linkou Chang Gung Memorial Hospital, Taoyuan, Taiwan; 4 Department of Pharmacy Systems, Outcomes and Policy, College of Pharmacy, University of Illinois at Chicago, Chicago, IL USA; 5 Liver Research Unit, Linkou Chang Gung Memorial Hospital and University, Taoyuan, Taiwan; 6 Section of Cardiology, Department of Medicine, Keelung Chang Gung Memorial Hospital, Keelung, Taiwan; 7 Chang Gung University College of Medicine, Taoyuan, Taiwan; 8 Department of Pharmaceutical Materials Management, Chang Gung Medical Foundation, Taoyuan, Taiwan; 9 Department of Pharmacy, National Cheng Kung University Hospital, Tainan, Taiwan

**Keywords:** Sodium glucose co-transporter 2 inhibitors, Dipeptidyl peptidase 4 inhibitors, Comparative effectiveness research, Multi-institutional electronic medical records, Real-world evidence

## Abstract

**Background:**

Sodium glucose cotransporter 2 (SGLT2) inhibitors have shown greater reductions of cardiovascular event risks than dipeptidyl peptidase-4 (DPP4) inhibitors, whereby possible mechanisms may involve the better pleiotropic effects of SGLT2 inhibitors. However, no published data are currently available to directly compare glycemic and pleiotropic effects in real-world type 2 diabetes patients initiating SGLT2 inhibitors or DPP4 inhibitors.

**Method:**

We conducted a retrospective cohort study by analyzing the Chang Gung Research Database, the largest multi-institutional electronic medical records database in Taiwan. We included patients newly receiving SGLT2 inhibitor or DPP4 inhibitor intensification therapy for type 2 diabetes from 2016 to 2017. We matched SGLT2 inhibitor users to DPP4 inhibitor users (1:4) by propensity scores to ensure comparable characteristics between the groups. We primarily evaluated 1-year post-treatment changes of hemoglobin A1c (HbA1c) after SGLT2 inhibitor or DPP4 inhibitor initiation, using two-tailed independent t-test. We also evaluated post-treatment changes in body weight, systolic blood pressure (SBP), alanine aminotransferase (ALT) and estimated glomerular filtration rate (eGFR) values, associated with SGLT2 inhibitors and DPP4 inhibitors.

**Results:**

We identified a cohort of 2028 SGLT2 inhibitors and 8112 matched DPP4 inhibitors new users. SGLT2 inhibitors and DPP4 inhibitors showed similar HbA1c reductions (− 1.0 vs. − 1.1%; P = 0.076), but patients receiving SGLT2 inhibitors had greater improvements in body weight (− 1.5 vs. − 1.0 kg; P = 0.008), SBP (− 2.5 vs. − 0.7 mmHg; P < 0.001) and ALT values (− 4.1 vs. − 0.0 U/l; P < 0.001) and smaller declines in eGFR values (− 2.0 vs. − 3.5 ml/min/1.73 m^2^; P < 0.001) when compared to DPP4 inhibitors.

**Conclusion:**

SGLT2 inhibitors had glucose-lowering effects comparable to those of DPP4 inhibitors but more favorable pleiotropic effects on body weight, ALT and eGFR changes, potentially improving type 2 diabetes patients’ cardio-metabolic disease risks.

## Background

Type 2 diabetes increases the risk of cardiovascular disease [1]. Control of glucose is considered first priority in the treatment of type 2 diabetes. Moreover, it is well known that maintaining appropriate body weight, blood pressure, and renal function of patients is also crucial for the reduction of cardiovascular risk in patients with type 2 diabetes [2–4]. Specifically, diabetes patients with liver disease due to metabolic abnormalities may be associated with risk of cardiovascular events [5]. In recent decades, several new drugs of different therapeutic classes have been introduced into diabetes treatment, but the use of sodium glucose cotransporter 2 (SGLT2) inhibitors and dipeptidyl peptidase-4 (DPP4) inhibitors has increased substantially [6], possibly because of their favorable side effect profiles.

The management of type 2 diabetes requires multi-factorial considerations beyond glycemic controls. As aforementioned, patients’ body weight, blood pressure, hepatic and renal functions may be associated with cardiovascular outcomes. SGLT2 inhibitors have shown greater reductions of cardiovascular event risks than DPP4 inhibitors [7–10], whereby possible mechanisms may involve the better pleiotropic effects of SGLT2 inhibitors. For example, meta-analyses of clinical trials have found SGLT2 inhibitors bring similar improvement in HbA1c, but better reductions of body weight and systolic blood pressure (SBP) compared to DPP4 inhibitors [11, 12]. Several individual clinical trials have also indicated SGLT2 inhibitors show better improvements in alanine aminotransferase (ALT) values and delayed declines of estimated glomerular filtration rate (eGFR) values when compared to DPP4 inhibitors [13, 14].

Current evidence predominantly from clinical trials is potentially not applicable to clinical practice, because real-world patients are often diverse and complex in their co-morbidities and concomitant medications [15]. Therefore, it is important to replicate findings from clinical trials with real-world data. To date, no published data are available to directly compare glycemic and pleiotropic effects in real-world type 2 diabetes patients initiating SGLT2 inhibitors or DPP4 inhibitors. In the present study, we analyzed Taiwan’s multi-institutional electronic medical records to compare head-to-head the glucose-lowering effects of SGLT2 inhibitors vs. DPP4 inhibitors in type 2 diabetes patients. Moreover, we also compared post-treatment changes in patients’ body weight, SBP, ALT and eGFR values, which may reflect an improvement in regard to patients’ cardio-metabolic disease risks.

## Method

### Study design and setting

We conducted a retrospective cohort study by analyzing data from Chang Gung Research Database (CGRD) from 2016 to 2018. The CGRD is Taiwan’s largest multi-institutional electronic medical records database, covering 1.3 million individuals (6% of Taiwan’s population). The data structures of CGRD have been described elsewhere [16]. Briefly, CGRD includes records of all visits to emergency rooms, ambulatory departments and hospitalizations from seven hospitals distributed nationwide across Taiwan [16]. The CGRD identifies diseases based on the International Classification of Diseases, Ninth Revision, Clinical Modification (ICD-9-CM) before 2016, and ICD-10-CM afterwards. The accuracy and validity of diagnostic codes of CGRD have been established [17, 18]. Notably, CGRD contains various laboratory data which afford the possibility of valid assessment of the treatment outcome [16, 19].

### Study population and follow-up

We included all type 2 diabetes patients aged 18 years and older newly receiving SGLT2 inhibitors (i.e., empagliflozin and dapagliflozin) or DPP4 inhibitors (i.e., alogliptin, linagliptin, saxagliptin, sitagliptin, vildagliptin) from 2016 to 2017. Because SGLT2 inhibitors and DPP4 inhibitors are used specifically for intensification therapy for type 2 diabetes, based on the Taiwan National Health Insurance reimbursement guidelines, the validity of type 2 diabetes diagnoses is good. We defined the index date as the first prescription date for SGLT2 inhibitors or DPP4 inhibitors based on a 1-year washout period. To ensure we had sufficient data to evaluate patients’ baseline condition, we excluded patients with no visit before or after the index date. We also excluded patients lacking baseline laboratory data for blood glucose levels (i.e., HbA1c), renal functions (i.e., estimated glomerular filtration rate; eGFR), SBP, body weight or liver functions (i.e., ALT), because we considered them to have not received routine medical care in our study hospitals.

We performed intent-to-treat analysis and followed up patients for 1 year from the index date, regardless of subsequent treatment changes after the SGLT2 inhibitor or DPP4 inhibitor initiations. To address the issue of non-adherence, discontinuation of SGLT2 inhibitors or DPP4 inhibitors and irregular follow-up, we performed as-treated analysis by excluding patients who discontinued SGLT2 inhibitor or DPP4 inhibitor treatment or were lost to follow-up before a year after index date. Patients without a refill of prescriptions for SGLT2 inhibitors or DPP4 inhibitors over 90 days during the follow-up period were considered discontinuations.

### Co-variables

We described patients’ baseline characteristics including age, sex, hospital levels and background anti-diabetes medications (i.e., metformin, sulfonylureas, pioglitazone and glucagon-like peptide-1 receptor agonists) and background cardiovascular medications (i.e., statin, and angiotensin-converting enzyme inhibitors/angiotensin receptor blockers). We collected information on co-morbidities related to cardiovascular diseases (i.e., hypertension, coronary heart disease, ischemic stroke and peripheral artery diseases), diabetes complications (i.e., retinopathy, nephropathy and neuropathy) and composites score (i.e., Charlson comorbidity index) within 1 year before the index date. Other co-morbidities and concomitant medications and corresponding definitions are described in Additional file 1:Table S1 and Table S2.

### Propensity score matching

**Fig. 1:**
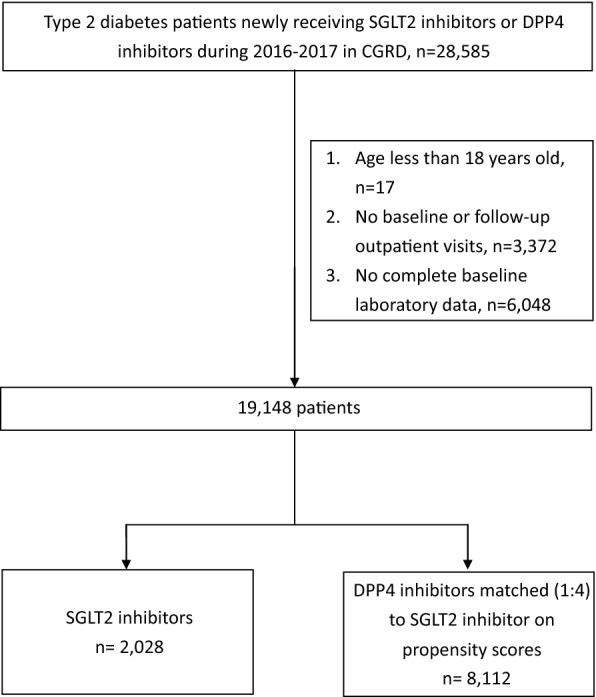
Patient selection flow chart. *CGRD* Chang Gung Research Database

To reduce possible channeling bias and to make a more homogeneous comparison, we used the propensity score method to generate comparable groups. The propensity scores were estimated for each treatment group by multivariable logistic regression models based on all baseline information listed in Table 1. We implemented a nearest neighbor matching algorithm that minimized distance within matched sets and applied a caliper of 0.05 on the propensity score scale with 8 → 1 greedy matching [20]. Four propensity score matched DPP4 inhibitor users were selected for each SGLT2 inhibitor user. We compare the baseline characteristics between the SGLT2 inhibitor and matched DPP4 inhibitor users in Table 1. The study cohort assembly is presented in Fig. 1.

**Table 1:** Baseline characteristics after propensity score matching

	SGLT2 inhibitors	DPP4 inhibitors	P value
Patients, n	2028	8112	
Age, mean years (SD)	60.9 (11.8)	61.3 (12.6)	0.285
Female, n (%)	1125 (55.5)	4617 (56.9)	0.241
HbA1c, mean % (SD)	8.7 (1.8)	8.6 (1.9)	0.063
Body weight, mean kg (SD)	71.6 (13.7)	70.9 (14.1)	0.070
SBP, mean mmHg (SD)	138.5 (19.9)	138.6 (20.5)	0.830
ALT, mean U/l (SD)	34.3 (32.2)	32.9 (31.2)	0.069
eGFR, mean ml/min/1.73 m^2^ (SD)	92.2 (29.4)	89.9 (39.0)	0.004
Hospital levels, n (%)			0.487
Medical centers	1116 (55.0)	4583 (56.5)	
Regional hospitals	537 (26.5)	2069 (25.5)	
District hospitals	375 (18.5)	1460 (18.0)	
Cardiovascular diseases, n (%)			
Hypertension	1335 (65.8)	5336 (65.8)	0.967
Hyperlipidemia	1312 (64.7)	5418 (66.8)	0.074
Coronary heart disease	396 (19.5)	1543 (19.0)	0.605
Peripheral artery disease	25 (1.2)	143 (1.8)	0.094
Heart failure	131 (6.5)	539 (6.6)	0.764
Ischemic stroke	91 (4.5)	355 (4.4)	0.828
Diabetes complications, n (%)			
Diabetic retinopathy	117 (5.8)	529 (6.5)	0.215
Diabetic neuropathy	159 (7.8)	670 (8.3)	0.538
Diabetic nephropathy	373 (18.4)	1516 (18.7)	0.760
Liver cirrhosis	54 (2.7)	165 (2.0)	0.082
Cancer	180 (8.9)	679 (8.4)	0.465
CCI score, mean (SD)	2.5 (1.6)	2.5 (1.6)	0.546
Previous hospitalization, n (%)	436 (21.5)	1637 (20.2)	0.188
Background anti-diabetes medications, n (%)			
Metformin	1691 (83.4)	6805 (83.9)	0.581
Sulfonylurea	887 (43.7)	3458 (42.6)	0.367
Glinide	49 (2.4)	187 (2.3)	0.767
Acarbose	203 (10.0)	780 (9.6)	0.591
Thiazolidinediones	122 (6.0)	466 (5.7)	0.640
Glucagon-like peptide-1 receptors antagonist	2 (0.1)	5 (0.1)	0.571
Insulin	331 (16.3)	1382 (17.0)	0.442
Background cardiovascular medications, n (%)			
Antiplatelet agents	602 (29.7)	2399 (29.6)	0.922
Beta blockers	516 (25.4)	1986 (24.5)	0.369
Angiotensin-converting enzyme inhibitors/angiotensin receptor blockers	1117 (55.1)	4432 (54.6)	0.720
Calcium channel blockers	794 (39.2)	3093 (38.1)	0.397
Diuretics	244 (12.0)	971 (12.0)	0.939
Statin	1156 (57.0)	4792 (59.1)	0.090
Fibrate	157 (7.7)	668 (8.2)	0.468
Ezetimibe	189 (9.3)	718 (8.9)	0.509

*ALT* alanine aminotransferase, *CCI* Charlson comorbidity index, *eGFR* estimated glomerular filtration rate, *SBP* systolic blood pressures

### Outcome measures

We evaluated the post-treatment changes in HbA1c after the 1 year follow-up period. Additionally, we compared the post-treatment changes in pleiotropic effects, including body weight, SBP, ALT and eGFR values between SGLT2 inhibitors and DPP4 inhibitors. We used the laboratory data nearest to the date of 1 year after index date as the post-treatment data. In most cases, patients were requested to visit a clinic and recheck their treatment outcomes and other laboratory parameters at intervals of less than 3 months. As a result, most laboratory data were retrieved within the last 3 months of the study period. The rates of missing values were low; for example, 0.0% and 11.3% of patients had no records of HbA1c and eGFR during the follow-up period, respectively. We implemented multiple imputations using the Markov chain Monte Carlo method with expectation–maximization algorithm [21], and combined 10 simulations to deal with missing follow-up outcome data. An overview of study design is presented in Additional file 1:Figure S1.

### Statistical analyses

We calculated mean ± standard deviation (SD) and number with proportion for continuous and categorized variables, respectively. We presented the changes of laboratory values before and after treatment by the means with 95% confidence interval (CI). We used paired t-tests to test the differences in laboratory value changes pre- and post-treatment within groups. We also used independent t-tests to examine the differences in the changes in laboratory values between groups. We considered statistical significance at two-tailed p value < 0.05. All statistical analyses were performed using SAS Enterprise version 5.1 (SAS Institute Inc., Cary, NC, USA).

### Subgroup analyses

We repeated the analysis within different subgroups to test the robustness of our study results. First, baseline glycemic controls and the number of prior anti-diabetes medication failures may influence the treatment response of diabetes [22, 23], so we conducted analyses to determine the effectiveness in patients with HbA1c > 7 and ≤ 7% or prior uses of > 1 and ≤ 1 anti-diabetes medications before the intensification therapy with SGLT2 inhibitors or DPP4 inhibitors. Second, we performed subgroup analyses in patients with normal (i.e., ALT ≤ 1× upper limit of normal, ULN) or abnormal liver functions (i.e., ALT > 1× ULN) to evaluate the treatment effectiveness in type 2 diabetes patients with different baseline liver functions. Third, we analyzed the comparative effectiveness in patients with eGFR > 60 and ≤ 60 ml/min/1.73 m^2^ because baseline renal functions might influence the treatment effects of SGLT2 inhibitors. Finally, type 2 diabetes patients were found to be clustered into distinct sub-populations based on BMI levels [24, 25], so we compared the treatment effectiveness in non-obese patients (i.e., body mass index, BMI < 27 kg/m^2^) and obese patients (i.e., BMI ≥ 27 kg/m^2^) [26].

## Results

We identified a total of 19,148 patients who initiated SGLT2 inhibitors or DPP4 inhibitors based on study inclusion and exclusion criteria (Fig. 1). We included all 2028 SGLT2 inhibitor new users and selected 8112 matched DPP4 inhibitor new users for the analysis. The baseline characteristics of the SGLT2 inhibitor and matched DPP4 inhibitor groups were comparable with mean ages of 60.9 ± 11.8 vs. 61.3 ± 12.6 years; and 55.5% vs. 56.9% female, respectively. The mean baseline HbA1c, body weight, SBP and ALT values for SGLT2 inhibitors and matched DPP4 inhibitor users were also similar (Hba1c: 8.7 ± 1.8% vs. 8.6 ± 1.9%; body weight: 71.6 ± 13.7 kg vs. 70.9 ± 14.1 kg; SBP: 138.5 ± 9.9 mmHg vs. 138.6 ± 20.5 mmHg; ALT: 34.3 ± 32.2 U/l vs. 32.9 ± 31.2 U/l). However, patients’ eGFR values were higher for SGLT2 inhibitors (92.2 ± 29.4 ml/min/1.73 m^2^) than matched DPP4 inhibitors (89.9 ± 39.9 ml/min/1.73 m^2^). Other baseline characteristics, such as co-morbidities and concomitant medications were well balanced between the SGLT2 inhibitor and matched DPP4 inhibitor groups (all p-values > 0.05) (Table 1).

**Fig. 2:**
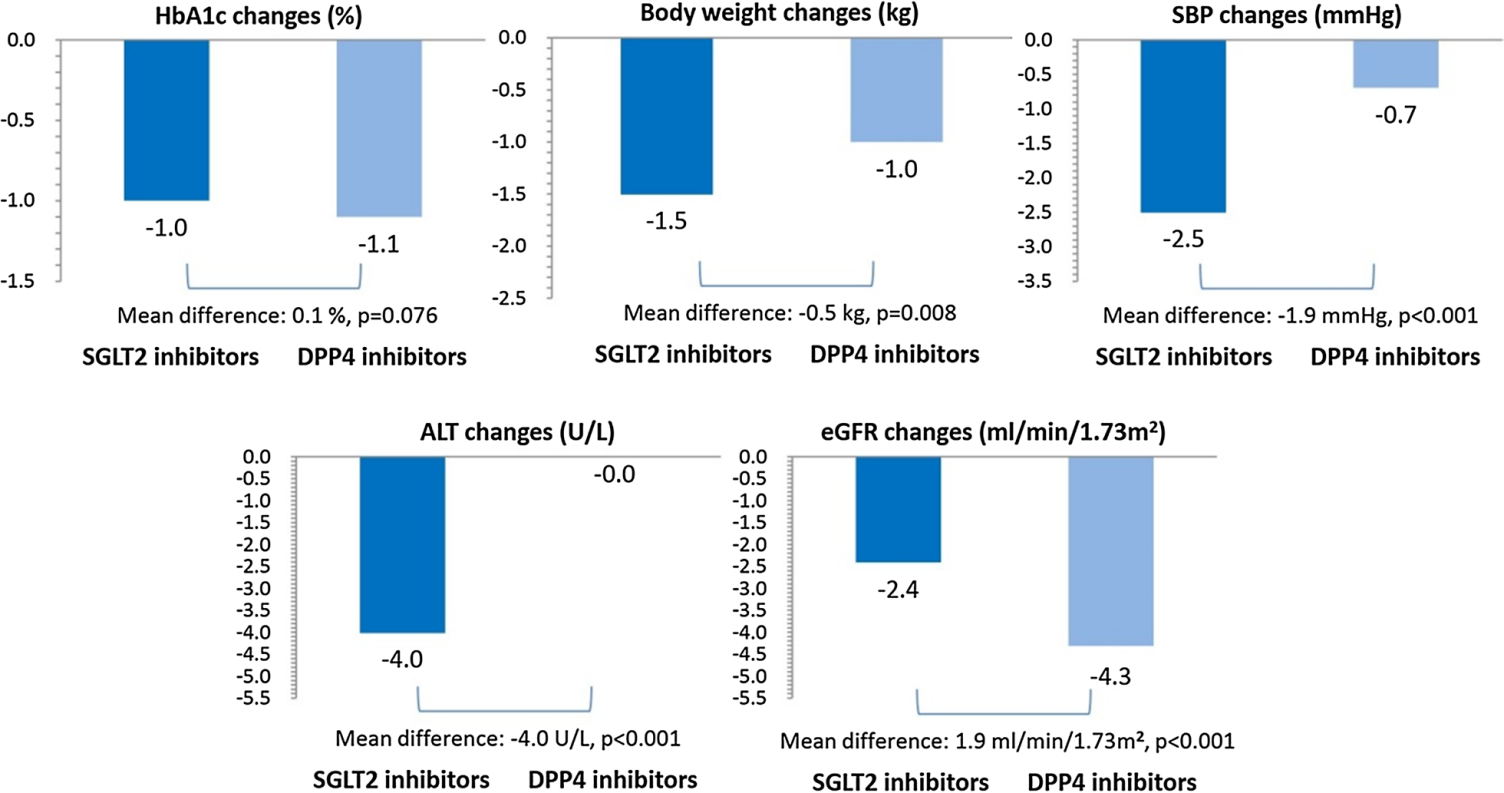
Comparisons of glycemic and pleiotropic effects between SGLT2 inhibitors and DPP4 inhibitors

Mean HbA1c levels for pre- and post-treatment are presented in Fig. 2. We found both SGLT2 inhibitors (− 1.0%; 95% CI − 1.10 to − 0.96) and matched DPP4 inhibitors (− 1.1%; 95% CI − 1.14 to − 1.07) decreased the HbA1c levels after 1-year intensification therapy. The reductions of HbA1c levels were similar between the SGLT2 inhibitor and matched DPP4 inhibitor groups (p = 0.076).

We present the changes in pleiotropic parameters after the SGLT2 inhibitor or matched DPP4 inhibitor treatment in Fig. 2. In patients receiving SGLT2 inhibitors, the body weight, SBP and ALT values were improved by − 1.5 kg (95% CI − 1.8 to − 1.2), − 2.5 mmHg (95% CI − 3.4 to − 1.6) and − 4.0 U/l (95% CI − 5.5 to − 2.6), respectively. In patients receiving DPP4 inhibitors, the body weight and SBP were also improved by − 1.0 kg (95% CI − 1.1 to − 0.8), − 0.7 mmHg (95% CI − 1.1 to − 0.2), respectively, but not the ALT values (− 0.0 U/l; 95% CI − 1.2 to 1.2). We found eGFR values were decreased in both SGLT2 inhibitor users (− 2.4 ml/min/1.73 m^2^; 95% CI − 3.2 to − 1.6) and DPP4 inhibitor users (− 4.3 ml/min/1.73 m^2^; 95% CI − 4.8 to − 3.7), but SGLT2 inhibitors led to less decline in eGFR values than DPP4 inhibitors. Compared to DPP4 inhibitors, SGLT2 inhibitors have more favorable pleiotropic effects on body weight (p = 0.008), SBP (p < 0.001), ALT and eGFR values (p < 0.001).

After we excluded patients who discontinued SGLT2 inhibitors or DPP4 inhibitors or were lost to follow-up before the end of the 1-year observational period, and repeated the analyses, the results remained consistent with the main analyses in that both SGLT2 inhibitors (− 1.2%; 95% CI − 1.30 to − 1.09) and DPP4 inhibitors (− 1.1%; 95% CI − 1.20 to − 1.07) decreased HbA1c levels and the changes were similar between the SGLT2 inhibitor and matched DPP4 inhibitor groups (p = 0.390) (Additional file 1:Table S3).

### Subgroup analyses

**Fig. 3:**
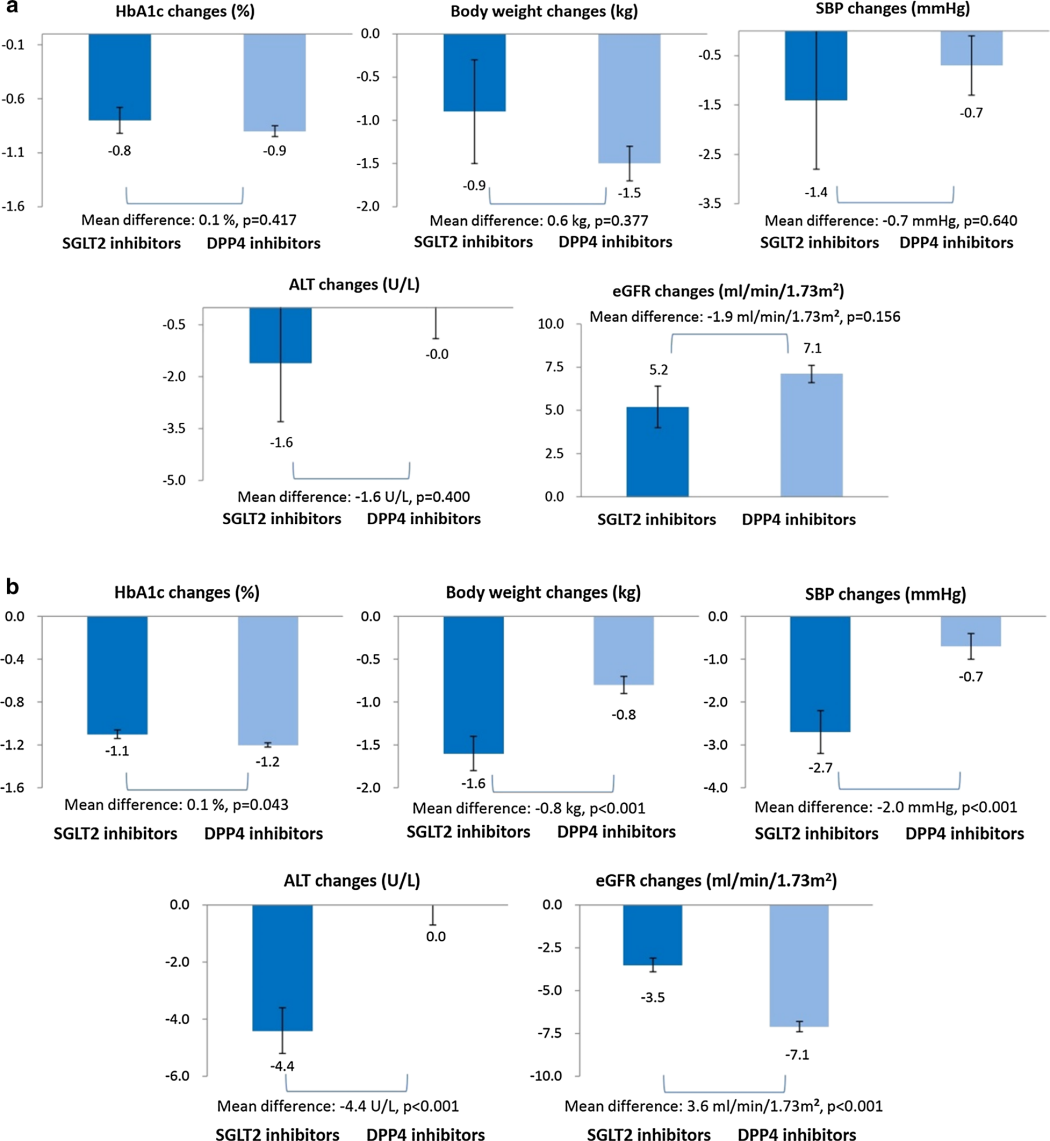
Comparisons of glycemic and pleiotropic effects between SGLT2 inhibitors and DPP4 inhibitors in patients with different eGFR levels. **a** Baseline eGFR < 60 ml/min/1.73m^2^. **b** Baseline eGFR ≥ 60 ml/min/1.73m^2^

The trends of subgroup analyses were mostly consistent with the main analysis (Additional file 1:Table S4). Specifically, we found SGLT2 inhibitors still had similar reduction effects on HbA1c levels compared to DPP4 inhibitors. We found the effects on body weight, SBP, ALT and eGFR values were better in SGLT2 inhibitors than DPP4 inhibitors throughout a series of subgroup analyses with HbA1c > 7 or ≤ 7%, ALT > 1× or ≤ 1× ULN, with or without a history of treatment failures of > 1 anti-diabetes medications, and with BMI ≥ 27 kg/m^2^ or < 27 kg/m^2^. Notably, the magnitude of change in ALT was higher in patients with baseline BMI ≥ 27 kg/m^2^ (− 6.3 U/l in SGLT2 inhibitors vs. − 0.4 U/l in DPP4 inhibitors) compared to BMI < 27 kg/m^2^ (− 1.9 U/l in SGLT2 inhibitors vs. 0.1 U/l in DPP4 inhibitors). However, in the subgroup of patients whose eGFR was less than 60 ml/min/1.73 m^2^, we found SGLT2 inhibitors did not provide significantly more beneficial pleiotropic effects than DPP4 inhibitors (Fig. 3).

## Discussion

The retrospective cohort study analyzed a large multi-institutional electronic medical records database in Taiwan to confirm the glycemic and pleiotropic effects of SGLT2 inhibitors in real-world practice. We compared SGLT2 inhibitors head-to-head with a matched cohort of patients receiving DPP4 inhibitors. We found patients who intensified therapy with SGLT2 inhibitors had similar glycemic controls, but more favorable pleiotropic effects on body weight, SBP, ALT and eGFR values, which might partly explain the better cardiovascular outcome, compared to DPP4 inhibitors [9].

### Glucose-lowering effects

Meta-analyses from the clinical trials have indicated the use of SGLT2 inhibitors is associated with better cardiovascular outcomes than DPP4 inhibitors [9]. Several large observational studies have also demonstrated more cardiovascular benefits in patients newly initiating SGLT2 inhibitors compared to DPP4 inhibitors [7, 8, 10]. SGLT2 inhibitors were associated with a significantly stronger reduction in HbA1c levels than were DPP4 inhibitors in clinical trials. However, DPP4 inhibitors produced better glucose-lowering responses in Asian populations due to the lower BMI [27], which warrants comparative clinical evaluations between SGLT2 inhibitors and DPP4 inhibitors in Asian patients. The differences in the reduction of HbA1c levels associated with SGLT2 inhibitors and DPP4 inhibitors used as add-on therapy to metformin in clinical trials were similar [11], and we found a similar result, whereby both SGLT2 inhibitors and DPP4 inhibitors reduced HbA1c levels by about 1.0% after 1 year of treatment, which might imply that the difference in favorable cardiovascular outcomes should be explained by other mechanisms.

### Body weight reductions

Weight control is known to be one of the key therapeutic goals in management of type 2 diabetes to reduce the cardiovascular disease risk [28], and modest weight reduction of as little as 5% can significantly improve cardiovascular disease risk [29]. Previous meta-analyses of clinical trials showed SGLT2 inhibitors led to significantly more weight loss compared to DPP4 inhibitors at ≥ 52 weeks (mean difference: − 2.5 kg, 95% CI − 2.8 to − 2.1) [30]. However, our study found fewer differences in body weight changes (− 0.5 kg) in real-world comparisons of SGLT2 inhibitors vs. DPP4 inhibitors. Potentially, the reason may be the complicated disease status and co-medications in real-world patients, which may affect the ability to achieve optimal weight reduction from SGLT2 inhibitors [31, 32]. Given our observations, the effects of SGLT2 inhibitors and DPP4 inhibitors on weight should be considered when individualizing type 2 diabetes therapy.

### Systolic blood pressure controls

About 75% of cardiovascular disease in diabetes may be attributable to hypertension, demanding clinical attention to patients with coexistent diabetes and hypertension [33]. SGLT2 inhibitors were associated with a greater decrease in SBP by 2.3–5.8 mmHg compared with DPP4 inhibitors in previous network meta-analyses of clinical trials [12]. In this study, we also found SGLT2 inhibitors reduce SBP by 1.9 mmHg more than DPP4 inhibitors in real-world patients. Probable reasons for better reduction of SBP may lie in the osmotic diuretic and mild natriuretic effects of SGLT2 inhibitors [34]. Because of this reduction in cardiovascular event risk by lowering SBP, our findings suggest that compared to DPP4 inhibitors, SGLT2 inhibitors as intensification therapy may provide a more valuable therapeutic option in type 2 diabetes patients.

### Alanine aminotransferase effects

Type 2 diabetes cases with reported 10% ALT abnormality are largely due to non-alcoholic fatty liver disease [35, 36]. Elevated ALT levels may increase two-fold the risk for cardiovascular disease and diabetes mortality [37]. Bajaj et al. [38] analyzed a large Canadian diabetes register and reported that 6-month SGLT2 inhibitor treatment could improve the ALT values while DPP4 inhibitors did not have significantly beneficial effects. Consistent with the previous finding, we extended the better ALT improvements associated with SGLT2 inhibitors vs. DPP4 inhibitors in type 2 diabetes patients with liver disease and 1-year follow-up. For example, we found that SGLT2 inhibitors could reduce the ALT values (− 8.9 U/l, 95% CI − 17.0 to − 0.9) while DPP4 inhibitors did not significantly improve the ALT (− 3.8 U/l, 95% CI − 15.8 to 8.2) in type 2 diabetes patients with liver cirrhosis after 1-year treatment (data not shown). The mechanism of better ALT improvement may be associated with more weight reduction through SGLT2 inhibitors than DPP4 inhibitors after our comparisons of glycemic and other pleiotropic effects. We found SGLT2 inhibitors could improve ALT values more in obese patients, compared to non-obese patients. This may reflect a greater effect on reduction of body weight and/or potential fatty liver in obese patients compared to non-obese patients [39]. In addition, anti-inflammatory effects and reduction of oxidative stress from SGLT2 inhibitors may also play an important role in the favorable ALT effects. Our findings suggest SGLT2 inhibitors might be more suitable for patients with unmet glycemic controls and ALT abnormality, compared to DPP4 inhibitors.

### Estimated glomerular filtration rate effects

Reduced kidney function in type 2 diabetes patients is the clinical indicator for poor cardiovascular outcomes and increased mortality [40]. Meta-analyses of clinical trials have demonstrated beneficial renal effects from SGLT2 inhibitors and DPP4 inhibitors [41, 42], but the underlying mechanism of renal protection is different between these two drug classes [43, 44]. For example, in addition to the better improvement of glycemic conditions, SGLT2 inhibitors could delay renal function deterioration by reduction of intraglomerular pressure [45]. Based on this unique mechanism, our findings support that SGLT2 inhibitors could better attenuate the eGFR declines after 1-year therapy, compared to DPP4 inhibitors. Given the totality of the outcome data, SGLT2 inhibitors may be preferable in type 2 diabetes patients with additional needs to reduce the risk of eGFR progression.

### Renal functions determine the effects of SGLT2 inhibitors

As SGLT2 inhibitors act by inhibiting reuptake of glucose and sodium filtered at the glomerulus, attenuation of clinical effects is to be expected with declining renal functions [46]. However, previous studies have shown conflicting results with regard to the effectiveness of SGLT2 inhibitors in patients with chronic kidney diseases [47, 48]. We found SGLT2 inhibitors had less glycemic and pleiotropic effects in type 2 diabetes patients with eGFR ≤ 60 ml/min/1.73 m^2^, compared to those with eGFR > 60 ml/min/1.73 m^2^. In addition, there were no significant differences between SGLT2 inhibitors and DPP4 inhibitors in the changes of body weight, SBP, ALT and eGFR in patients with eGFR ≤ 60 ml/min/1.73 m^2^, which indicates that renal functions may be the key point to determine the pleiotropic effects of SGLT2 inhibitors vs. DPP4 inhibitors. Our study indicated both SGLT2 inhibitors and DPP4 inhibitors could slightly improve patients’ renal functions in patients with eGFR < 60 ml/min/1.73 m^2^. The findings were consistent with previous studies on SGLT2 inhibitors and DPP4 inhibitors [47, 49]. The mechanisms of improving renal functions could be complex. In addition to direct effects from anti-diabetes medications, one possible explanation is that better glycemic controls after treatment may preserve or improve patients’ renal functions [50, 51]. Moreover, the improvements of renal functions could also be attributed to lifestyle modifications as a result of education provided by healthcare professionals. Interestingly, we found a slight difference without statistical significance in the changes of eGFR values between SGLT2 inhibitors and DPP4 inhibitors. However, there were only limited numbers of patients with eGFR < 60 ml/min/1.73 m^2^ in our study, so future investigations with more patients with chronic kidney diseases are required to provide more conclusive evidence.

### Strengths and limitations

We included real-world patients with diverse conditions such as chronic liver diseases or renal diseases to confirm the findings from clinical trials. The subgroup analyses by patients’ ALT and eGFR levels provided better understanding of the use of SGLT2 inhibitors in real-world practice. The CGRD contains a large-size representative sample with standardized and relevant clinical information for analyses. However, like with all retrospective cohort study, some limitations of the study should be noted. First, because clinicians may prefer to use SGLT2 inhibitors for higher cardiovascular or renal event risk over DPP4 inhibitors, confounding by indications should be considered. We performed propensity score matching to balance patients’ characteristics between SGLT2 inhibitors and DPP4 inhibitors and to minimize bias. We also stratified patients by baseline glycemic controls for more homogeneous group comparisons. Second, we only evaluated the changes in two frequently measured indicators, ALT and eGFR values, representing the liver and kidney function, respectively. Third, recent studies have proven the SGLT2 inhibitors could increase levels of ketone, arginine, arginine/asymmetric dimethylarginine (ADMA) ratio and improve the left ventricular diastolic function which may provide favorable effects on cardiovascular outcomes [52–55]. Although supporting evidence is still lacking, the changes in ketone and arginine/ADMA levels from SGLT2 inhibitors presumably vary in patients with different renal functions. The aforementioned observations offer alternative etiological mechanisms for favorable cardiovascular outcomes in SGLT2 inhibitors compared to DPP4 inhibitors. They might also explain why patients’ baseline renal function is one of the key factors in achieving favorable effects of SGLT2 inhibitors. Since we were not able to capture these laboratory parameters in the study, and they are not monitored in routine care, we encourage future analysis collecting the information to confirm the hypothesis. Fourth, patients may have been lost to follow-up as they transferred to hospitals other than CGRD. However, we found the loss to follow-up and discontinuation rates did not differ between SGLT2 inhibitors and DPP4 inhibitors and that bias could be eliminated from the comparisons. The results from as-treated analyses excluding patients who discontinued the drugs remained consistent with the main findings. Fifth, we did not include canagliflozin new users since it was not available until July 2018 in CGRD, so our findings cannot be generalized to canagliflozin. Sixth, this study was based on changes in laboratory data between two drug classes, and thus no inference on cardiovascular events could be made. Nevertheless, we conducted a post hoc analysis and investigated the incidence rate of major cardiovascular events (MACE), including myocardial infarction, ischemic stroke and cardiovascular death, because SGLT2 inhibitors have been proven to reduce MACE in placebo-controlled trials [56–59]. We found the incidence rate of MACE was 12.6 per 1000 person-years in SGLT2 inhibitors versus 14.5 per 1000 person-years in DPP4 inhibitors. The findings provide a foundation for future study comparing the risk of cardiovascular events between SGLT2 inhibitors and DPP4 inhibitors.

## Conclusion

Our head-to-head comparisons indicate that SGLT2 inhibitors provide similar glycemic controls compared with DPP4 inhibitors, but have better effects on body weight, SBP, ALT and eGFR changes after one-year treatment in type 2 diabetes patients. The study establishes a clinical ground for future prospective studies to confirm favorable pleiotropic effects of SGLT2 inhibitors and their associated cardiovascular outcomes.

## Supplementary information

**Additional file 1: Figure S1.** Overview of Study design. **Table S1.** ICD codes for co-morbidity. **Table S2.** List for co-medication. **Table S3.** As-treated analyses of the changes in clinical parameters from the baseline to 1 year post treatment. **Table S4.** Subgroup analyses of the changes in clinical parameters from the baseline to 1 year post treatment.

## Data Availability

Data sharing is not applicable to this study as data management and analysis were performed on a statistics server through remote access in Chang Gung Medical Foundation in Taiwan, for privacy and safety concerns.

## Abbreviations

ALT: alanine aminotransferase
BMI: body mass index
CGRD: Chang Gung Research Database
CI: confidence intervals
DPP4: dipeptidyl peptidase-4
eGFR: estimated glomerular filtration rate
HbA1c: hemoglobin A1c
ICD-9-CM: International Classification of Diseases, Ninth Revision, Clinical Modification
ICD-10-CM: International Classification of Diseases, Tenth Revision, Clinical Modification
SBP: systolic blood pressure
SD: standard deviation
SGLT2v: sodium glucose cotransporter 2
ULN: upper limit of normal
