# Supplementary material for: Favorable pleiotropic effects of sodium glucose cotransporter 2 inhibitors: head-to-head comparisons with dipeptidyl peptidase-4 inhibitors in type 2 diabetes patients
Source: Cardiovasc Diabetol. 2020 Feb 12;19:17. doi: 10.1186/s12933-020-0990-2 (PMC7014757; doi:10.1186/s12933-020-0990-2)
Supplement: Supplementary file 1 — Additional file 1: Figure S1. Overview of Study design. Table S1. ICD codes for co-morbidity. Table S2. List for co-medication. Table S3. As-treated analyses of the changes in clinical parameters from the baseline to 1 year post treatment. Table S4. Subgroup analyses of the changes in clinical parameters from the baseline to 1 year post treatment. [file 12933_2020_990_MOESM1_ESM.pdf]

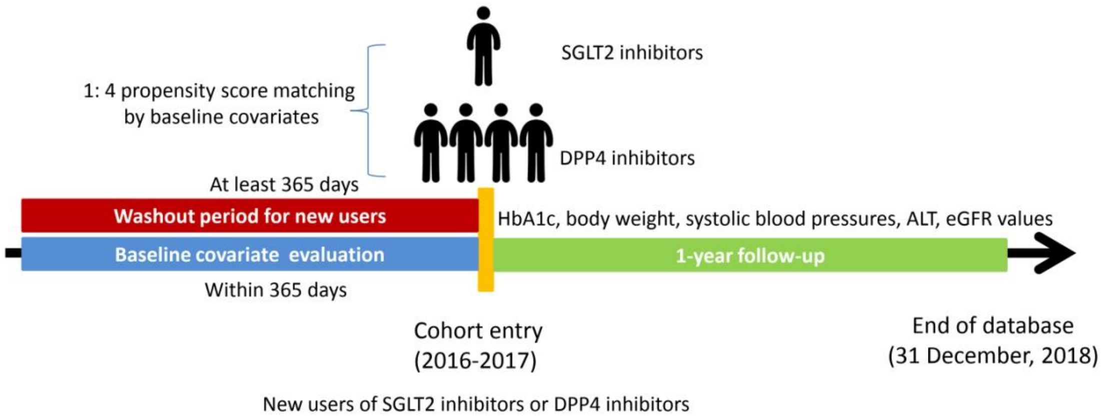

Figure S1. Overview of Study design

**Table S1.** ICD codes for co-morbidity		
Diseases	ICD-9 codes	ICD-10 codes
Hypertension	401, 402, 403, 404, 405	I10, I11, I12, I13, I15, I16
Hyperlipidemia	272	E78
Coronary heart disease	410, 411, 412, 413, 414	I20, I21, I22, I23, I24, I25
Peripheral artery disease	440	I70, I73
Heart failure	428	I50
Ischemic stroke	433,434	I63
Diabetic retinopathy	2505	E083, E113
Diabetic neuropathy	2506	E084, E114
Diabetic nephropathy	2504	E082, E112
Liver cirrhosis	5712, 5715	K702, K7030, K7031, K741, K742, K7460, K7469
Cancer	140-239	C0-D4

**Table S2.** List for co-medication	
Drug class	Drug name
Anti-platelet agents	Aspirin, clopidogrel, dipyridamole, ticagrelor
Beta blocker	Atenolol, bisoprolol, carvedilol, metoprolol, propranolol,
Angiotensin-converting enzyme inhibitors or angiotensin receptor blockers	Azilsartan, candestartan, captopril, enalapril, fosinopril, irbesartan, losartan, olmesartan, ramipril telmisartan, valsartan
Calcium channel blockers	Amlodipine, diltiazem, felodipine, lercanidipine, nifedipine, verapamil
Diuretics	Acetazolamide, Amiloride, Benzyl hydrochlorothiazide, Bumetanide, Furosemide, Hydrochlorothiazide, Indapamide, Spironolactone
Statin	Atorvastatin, fluvastatin, pitavastatin, rosuvastatin, simvastatin
Fibrate	Fenofibrate, gemfibrozil
Sulfonylurea	Glipizide, gliclazide, glimepiride, glyburide
Thiazolidinediones	Pioglitzone
Glinides	Nateglinide, meglitinide, repaglinde
Glucagon-like peptide-1 receptor antagonists	Dulaglutide, exenatide, liraglutide
Insulin	Rapid, short, intermediate and long-acting insulins

Table S3. As-treated analyses of the changes in clinical parameters from the baseline to 1 year post treatment

	SGLT2 inhibitors	DPP4 inhibitors	P value
HbA1c, % (SD)			
Pre-treatment	8.8±1.7	8.4±1.8	<0.001
Post-treatment	7.6±1.1	7.3±1.2	<0.001
Changes from baseline	-1.2±1.5	-1.1±1.8	0.390
Body weight, kg (SD)			
Pre-treatment	72.0±13.0	70.5±13.5	0.004
Post-treatment	70.4±12.6	69.8±12.6	0.214
Changes from baseline	-1.7±6.8	-0.8±7.7	0.001
SBP, mmHg (SD)			
Pre-treatment	139.4±19.6	138.9±20.3	0.537
Post-treatment	135.4±19.2	138.6±20.4	<0.001
Changes from baseline	-4.0±19.7	-0.3±21.7	<0.001
ALT, U/L (SD)			
Pre-treatment	33.9±27.2	32.3±34.7	0.148
Post-treatment	29.8±23.8	31.1±26.9	0.170
Changes from baseline	-4.2±27.5	-1.2±35.7	0.011
eGFR, ml/min/1.73m^2 (SD)			
Pre-treatment	93.7±28.2	87.7±36.8	<0.001
Post-treatment	91.0±27.7	83.7±32.8	<0.001
Changes from baseline	-2.7±17.8	-4.0±20.9	0.085

**Table S4.** Subgroup analyses of the changes in clinical parameters from the baseline to 1 year post treatment			
	SGLT2 inhibitors	DPP4 inhibitors	P value
Patients with HbA1c >7%	N=1,698	N=6,700	
HbA1c, % (SD)			
Pre-treatment	9.1±1.7	9.0±1.8	0.182
Post-treatment	7.8±1.3	7.6±1.3	<0.001
Changes from baseline	-1.3±1.7	-1.4±1.9	0.018
Body weight, kg (SD)			
Pre-treatment	71.2±13.5	71.1±14.1	0.771
Post-treatment	69.8±12.8	70.2±12.9	0.300
Changes from baseline	-1.4±7.8	-0.9±8.1	0.027
SBP, mmHg (SD)			
Pre-treatment	139.2±20.2	138.9±20.6	0.644
Post-treatment	136.2±19.6	138.2±20.2	<0.001
Changes from baseline	-3.1±20.4	-0.7±22.2	<0.001
ALT, U/L (SD)			
Pre-treatment	34.7±33.6	33.7±31.0	0.254
Post-treatment	30.0±23.4	33.3±44.8	<0.001
Changes from baseline	-4.6±34.0	-0.4±47.6	<0.001
eGFR, ml/min/1.73m^2 (SD)			
Pre-treatment	92.5±29.0	92.0±38.9	0.536
Post-treatment	90.1±29.3	87.1±33.5	<0.001
Changes from baseline	-2.4±17.7	-4.9±25.8	<0.001
Patients with HbA1c ≤7%	N=320	N=1,412	
HbA1c, % (SD)			
Pre-treatment	6.5±0.4	6.5±0.5	0.348
Post-treatment	6.8±0.8	6.7±1.0	0.633
Changes from baseline	0.2±0.78	0.2±1.0	0.994
Body weight, kg (SD)			
Pre-treatment	73.5±14.3	70.3±14.2	<0.001
Post-treatment	71.6±13.3	69.2±13.1	0.004
Changes from baseline	-1.9±6.8	-1.1±8.1	0.073
SBP, mmHg (SD)			
Pre-treatment	134.6±17.8	136.8±19.9	0.055
Post-treatment	134.9±18.0	136.5±20.6	0.182
Changes from baseline	0.2±20.2	-0.3±21.9	0.690
ALT, U/L (SD)			
Pre-treatment	32.6±24.2	29.4±31.6	0.047
Post-treatment	31.8±27.7	31.0±76.6	0.769
Changes from baseline	-0.8±25.4	1.6±79.2	0.336
eGFR, ml/min/1.73m^2 (SD)			
Pre-treatment	90.1±31.5	79.9±38.0	<0.001
Post-treatment	87.7±29.5	78.5±34.4	<0.001
Changes from baseline	-2.4±22.3	-1.4±24.6	0.453
Patients with >1 anti-diabetes medication at	N=1,116	N=4,208	

baseline			
HbA1c, % (SD)			
Pre-treatment	9.1±1.8	9.0±2.1	0.091
Post-treatment	7.9±1.4	7.7±1.4	<0.001
Changes from baseline	-1.2±1.8	-1.3±2.1	0.469
Body weight, kg (SD)			
Pre-treatment	70.0±13.0	69.5±13.6	0.238
Post-treatment	68.7±12.4	68.9±12.6	0.508
Changes from baseline	-1.4±6.5	-0.5±7.7	<0.001
SBP, mmHg (SD)			
Pre-treatment	138.8±20.2	138.4±20.7	0.475
Post-treatment	136.3±19.5	138.1±20.7	0.008
Changes from baseline	-2.6±20.4	-0.2±22.4	0.001
ALT, U/L (SD)			
Pre-treatment	33.9±35.9	30.9±28.0	0.010
Post-treatment	29.2±23.1	32.3±62.1	0.008
Changes from baseline	-4.6±35.5	1.5±63.8	<0.001
eGFR, ml/min/1.73m^2 (SD)			
Pre-treatment	91.4±29.4	90.6±40.7	0.442
Post-treatment	88.5±29.5	85.4±33.9	0.002
Changes from baseline	-2.8±18.2	-5.2±28.1	<0.001
Patients with ≤1 anti-diabetes medication at baseline	N=912	N=3,904	
HbA1c, % (SD)			
Pre-treatment	8.1±1.7	8.1±1.6	0.952
Post-treatment	7.3±1.1	7.2±1.1	0.002
Changes from baseline	-0.8±1.4	-0.9±1.6	0.012
Body weight, kg (SD)			
Pre-treatment	73.5±14.2	72.5±14.5	0.071
Post-treatment	71.8±13.3	71.1±13.3	0.164
Changes from baseline	-1.6±8.8	-1.4±8.4	0.556
SBP, mmHg (SD)			
Pre-treatment	138.0±19.4	138.8±20.2	0.270
Post-treatment	135.5±19.2	137.7±19.8	0.003
Changes from baseline	-2.5±20.3	-1.1±21.9	0.072
ALT, U/L (SD)			
Pre-treatment	34.9±27.1	35.1±34.1	0.839
Post-treatment	31.6±25.4	33.6±37.5	0.063
Changes from baseline	-3.3±29.2	-1.6±42.1	0.165
eGFR, ml/min/1.73m^2 (SD)			
Pre-treatment	93.1±29.5	89.2±37.1	<0.001
Post-treatment	91.0±29.2	85.9±33.6	<0.001
Changes from baseline	-1.9±18.9	-3.3±22.7	0.059
Patients with ALT ≤ 1X ULN	N=1591	N=6529	
HbA1c, % (SD)			

Pre-treatment	8.7±1.9	8.5±2.0	<0.001
Post-treatment	7.7±1.3	7.5±1.3	<0.001
Changes from baseline	-1.0±1.7	-1.1±1.9	0.164
Body weight, kg (SD)			
Pre-treatment	70.6±13.2	70.1±13.6	0.235
Post-treatment	69.3±12.6	69.4±12.5	0.767
Changes from baseline	-1.3±7.9	-0.8±7.9	0.024
SBP, mmHg (SD)			
Pre-treatment	138.7±20.0	138.3±20.5	0.480
Post-treatment	136.2±19.5	137.9±20.7	0.002
Changes from baseline	-2.6±20.5	-0.4±22.3	<0.001
ALT, U/L (SD)			
Pre-treatment	23.6±9.2	23.1±9.3	0.073
Post-treatment	26.1±19.8	27.8±49.0	0.026
Changes from baseline	2.5±19.9	4.7±48.8	0.005
eGFR, ml/min/1.73m^2 (SD)			
Pre-treatment	90.6±29.5	87.5±38.4	<0.001
Post-treatment	88.1±29.5	83.6±34.2	<0.001
Changes from baseline	-2.5±18.4	-3.9±24.1	0.007
Patients with ALT >1X ULN	N=437	N=1,583	
HbA1c, % (SD)			
Pre-treatment	8.5±1.7	8.7±1.8	0.108
Post-treatment	7.4±1.2	7.4±1.3	0.750
Changes from baseline	-1.1±1.6	-1.2±1.9	0.185
Body weight, kg (SD)			
Pre-treatment	75.1±14.6	74.2±15.8	0.258
Post-treatment	73.1±13.6	72.6±14.3	0.538
Changes from baseline	-2.2±6.4	-1.6±8.7	0.132
SBP, mmHg (SD)			
Pre-treatment	137.5±19.4	139.6±20.1	0.052
Post-treatment	135.3±18.8	137.9±18.6	0.013
Changes from baseline	-2.1±19.8	-1.7±21.7	0.735
ALT, U/L (SD)			
Pre-treatment	73.5±50.6	73.4±50.8	0.963
Post-treatment	45.6±31.6	53.8±57.1	<0.001
Changes from baseline	-27.9±53.4	-19.6±69.8	0.008
eGFR, ml/min/1.73m^2 (SD)			
Pre-treatment	98.0±28.6	99.7±40.0	0.315
Post-treatment	95.5±28.2	93.8±30.6	0.263
Changes from baseline	-2.3±19.1	-5.7±31.1	0.004
Patients with BMI ≥27 kg/m^2	N=987	N=3,515	
HbA1c, % (SD)			
Pre-treatment	8.5±1.7	8.5±1.8	0.428

Post-treatment	7.4±1.3	7.5±1.3	0.235
Changes from baseline	-1.0±1.6	-1.1±1.8	0.086
Body weight, kg (SD)			
Pre-treatment	80.4±11.7	80.9±12.7	0.190
Post-treatment	77.1±12.3	77.5±12.8	0.359
Changes from baseline	-3.2±7.8	-3.4±9.0	0.495
SBP, mmHg (SD)			
Pre-treatment	140.9±18.4	141.7±19.9	0.237
Post-treatment	137.5±19.6	140.4±19.8	<0.001
Changes from baseline	-3.4±20.5	-1.3±22.3	<0.001
ALT, U/L (SD)			
Pre-treatment	38.9±36.5	37.1±28.9	0.145
Post-treatment	32.6±24.6	36.7±60.1	0.002
Changes from baseline	-6.3±36.6	-0.4±59.4	<0.001
eGFR, ml/min/1.73m^2 (SD)			
Pre-treatment	92.8±28.2	88.0±37.6	<0.001
Post-treatment	90.6±27.9	84.3±32.1	<0.001
Changes from baseline	-2.2±17.7	-3.5±25.5	0.066
Patients with BMI <27 kg/m^2	N=964	N=4,102	
HbA1c, % (SD)			
Pre-treatment	8.8±1.8	8.6±2.0	0.001
Post-treatment	7.8±1.3	7.5±1.3	<0.001
Changes from baseline	-1.1±1.7	-1.1±1.9	0.266
Body weight, kg (SD)			
Pre-treatment	62.8±9.0	62.6±9.1	0.499
Post-treatment	63.0±9.4	63.7±9.5	0.024
Changes from baseline	0.1±6.7	1.1±6.2	<0.001
SBP, mmHg (SD)			
Pre-treatment	136.1±20.4	136.0±20.5	0.935
Post-treatment	134.4±18.8	135.8±21.9	0.036
Changes from baseline	-1.7±20.1	-0.2±21.9	0.034
ALT, U/L (SD)			
Pre-treatment	29.9±27.2	29.8±33.6	0.942
Post-treatment	28.0±23.6	29.9±45.4	0.062
Changes from baseline	-1.9±29.1	0.1±52.2	0.105
eGFR, ml/min/1.73m^2 (SD)			
Pre-treatment	91.8±30.6	91.1±38.8	0.569
Post-treatment	88.7±30.7	86.5±34.5	0.047
Changes from baseline	-3.0±19.0	-4.7±23.6	0.016
